# Enhancing the performance of a mutant pyrrolysyl-tRNA synthetase to create a highly versatile eukaryotic cell-free protein synthesis tool

**DOI:** 10.1038/s41598-023-42198-8

**Published:** 2023-09-14

**Authors:** Jeffrey L. Schloßhauer, Anne Zemella, Srujan K. Dondapati, Lena Thoring, Manpreet Meyer, Stefan Kubick

**Affiliations:** 1 Fraunhofer Project Group PZ-Syn of the Fraunhofer Institute for Cell Therapy and Immunology (IZI), Branch Bioanalytics and Bioprocesses (IZI-BB), Institute of Biotechnology,, Brandenburg University of Technology Cottbus-Senftenberg, Am Mühlenberg, Potsdam, Germany; 2 Fraunhofer Institute for Cell Therapy and Immunology (IZI), Branch Bioanalytics and Bioprocesses (IZI-BB), Am Mühlenberg, Potsdam, Germany; 3 Faculty of Health Sciences, Joint Faculty of the Brandenburg University of Technology Cottbus –Senftenberg, The Brandenburg Medical School Theodor Fontane, University of Potsdam, Potsdam, Germany; 4 Laboratory of Protein Biochemistry, Institute for Chemistry and Biochemistry, Freie Universität Berlin, Thielallee 63, 14195 Berlin, Germany

**Keywords:** Biochemistry, Chemical biology, Physiology, Systems biology

## Abstract

Modification of proteins with a broad range of chemical functionalities enables the investigation of protein structure and activity by manipulating polypeptides at single amino acid resolution. Indeed, various functional groups including bulky non-canonical amino acids like strained cyclooctenes could be introduced by the unique features of the binding pocket of the double mutant pyrrolysyl-tRNA synthetase (Y306A, Y384F), but the instable nature of the enzyme limits its application in vivo. Here, we constructed a cell-free protein production system, which increased the overall enzyme stability by combining different reaction compartments. Moreover, a co-expression approach in a one-pot reaction allowed straightforward site-specific fluorescent labeling of the functional complex membrane protein cystic fibrosis transmembrane conductance regulator. Our work provides a versatile platform for introducing various non-canonical amino acids into difficult-to-express proteins for structural and fluorescence based investigation of proteins activity.

## Introduction

**Figure 1:**
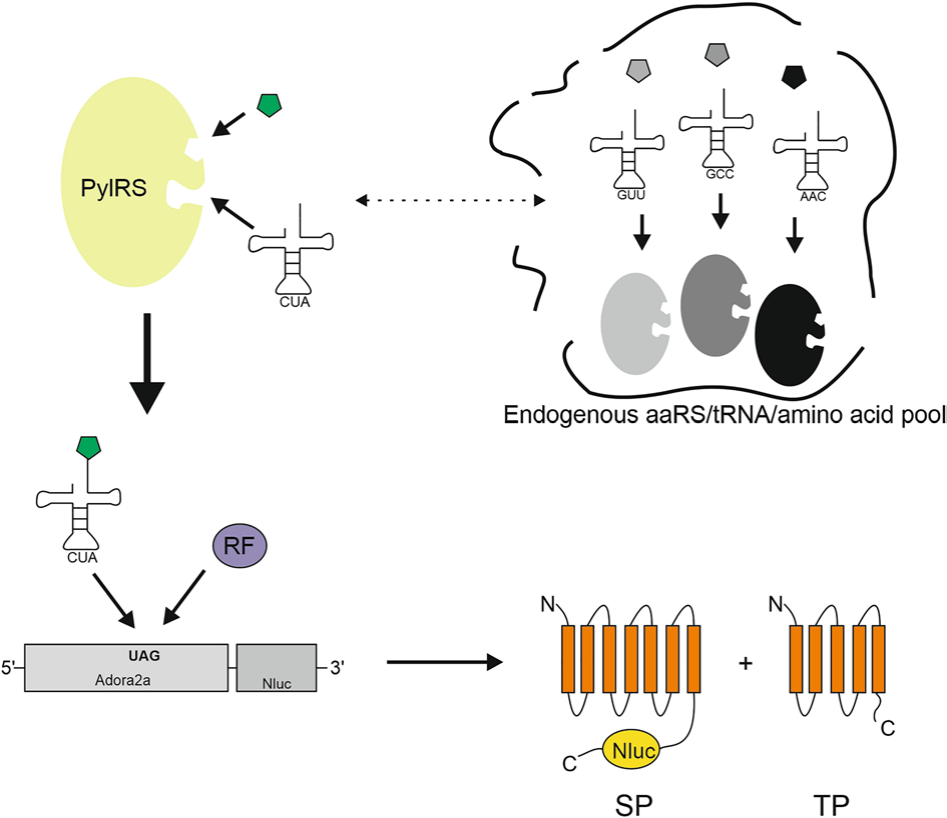
Principle of amber suppression based on PylRS-AF/tRNA(Pyl). The orthogonal PylRS-AF aminoacylates the non-canonical amino acid (green) to the tRNA(Pyl), without cross-reacting with endogenous components from the cell lysate (indicated by a dashed line). The aminoacylated tRNA(Pyl) competes with the release factor (RF) for the amber stop codon recognition sequence UAG. Full-length suppression product (SP) results in Nluc activity, while terminated Adora2a is a truncated termination product (TP).

Equipping proteins with new functional groups allows the investigation of protein interactions, folding, dynamics and localization. Various techniques exist to modify protein molecules with specific moieties, including fluorescent proteins, Intein-Extein ligation, Sortase A ligation, lysine modification as well as expressed chemical ligation^1–4^. Although, these technologies are widely used, there are many limitations. The large size of fluorescent fusion proteins like GFP can impact protein function and correct folding^5^. In addition, the mentioned methods are restricted to N- or C-terminal ligation, cysteine residues and motifs required for ligation remain in the polypeptide sequence^6^. While lysines are frequently used to modify proteins, it is difficult to modify lysines in a controlled and selective manner^7^. Alternatively, modifications of defined amino acid residues by substitution with chemical groups with diverse reactivity or biophysical properties would enhance protein examination. Thus, amber stop codon suppression provides the opportunity to incorporate a wide range of non-canonical amino acids (ncaa) with novel properties^8^. The basis of this technology is composed of an altered aminoacyl tRNA-synthetase (aaRS), which covalently couples a ncaa to an appropriate tRNA with an anticodon that is complementary to the amber stop codon^9^. During protein translation, the charged aminoacylated tRNA will be recognized by the translational machinery, leading to ncaa incorporation into the nascent polypeptide at the desired amber stop codon position. The system requires that aaRS, tRNA and cognate ncaa must not cross-react with endogenous aaRS, tRNAs and amino acids in the given protein production platform and are so called orthogonal components (Fig. 1)^10^.

Orthogonal translation based on *Methanosarcina mazei* pyrrolysyl-tRNA synthetase (*Mm*PylRS) and its cognate tRNA(Pyl) is of main interest, since it can be utilized in prokaryotic and eukaryotic cells and as well in whole animals^11^. More than 100 diverse ncaa could be introduced into proteins by the PylRS/tRNA(Pyl) pair, indicating the versatile utility^12^. Hence, the system was applied for the generation of photo-cross-linked, spin-labeled and fluorescent proteins^13–15^. An engineered mutant PylRS with a structure based mutation Y306A was reported to expand the binding pocket of the PylRS and a further mutation of a tyrosine residue to phenylalanine (Y384F) increased the suppression efficiency in vivo^16^. The major advantage of this double mutant PylRS-AF is the accommodation of large and bulky amino acids. Accordingly, proteins can be modified with strained cyclooctenes and –octynes^17^. Subsequent inverse electron demand Diels–Alder reactions, also named tetrazine ligations between a tetrazine group acting as diene and a dienophile such as strained cyclooct-2-yn-1-methylcarbamate lysine (SCO) can be accomplished^18^. Tetrazine ligation is non-toxic, does not require a catalyst, is highly biorthogonal and is multiple orders of magnitude faster than other click reactions with second order rate constants of up to 10^6^ M^−1^ s^−1^^19^. In contrast, other widely used click reactions, such as staudinger ligation and copper-catalyzed azido–alkyne cycloadditions are either slow or display cytotoxic effects in vivo^20^. Consequently, the unique properties of PylRS-AF and the given scope of possibilities strengthened us to develop a PylRS-AF based translation system. Therefore, the open environment of cell-free protein synthesis reactions ensures optimal requirements to manipulate protein translation^21^. The ncaa can be added in desired amounts directly to the reaction, thereby overcoming limitations of in vivo based protein labeling, where the ncaa must cross the cell membrane by endogenous amino acid transporters or diffusion^22^. The ncaa required for orthogonal translation must be offered in the cell culture medium and can lead to cell growth inhibition and increased cell toxicity^23^. Additionally, site-specific introduction of ncaa in cell-free reactions can be achieved within a few hours using a batch based format, while cell-based applications might take several days or weeks^24^.

To date, different in vitro protein production systems exist^25^. Translationally active lysates originated from *Escherichia coli* are widely applied and it was previously shown that site-specific labeling of diverse proteins can be achieved by altered PylRS variants in *E.coli* cell-free protein synthesis^26–28^. However, post-translational modifications (PTM) are limited in *E.coli* based approaches. Indeed, PTMs are feasible in eukaryotes, but in vivo production of complex membrane proteins is often associated with overexpression^29^. As a consequence, the cell membrane integrity of other host proteins can be negatively influenced^30^. To this end, eukaryotic cell lysates arose from Chinese hamster ovary (CHO) cells and *Spodoptera frugiperda* 21 *(Sf*21) cells were shown to translocate complex membrane proteins into a lipid bilayer derived from the endoplasmic reticulum (ER) in a co-translational manner^31,32^. Preliminary studies showed simultaneously integration of the G protein-coupled receptor adenosine A2a (Adora2a) into these ER-derived microsomal structures in CHO based cell-free reactions and fluorescent labeling when utilizing an evolved *E.coli* tyrosyl tRNA-synthetase^33^. Moreover, co- and post-translational modifications including glycosylation, lipidation, phosphorylation and disulfide bonds can be realized, thus enhancing the production of complex proteins, such as the cystic fibrosis transmembrane conductance regulator (CFTR)^34^. This large membrane protein is composed of 12 transmembrane helices. In epithelial cells CFTR is essential for conductance of chloride, bicarbonate and thiocyanate^35,36^. It was reported that folding of the anion channel CFTR is impaired by the deletion of phenylalanine at position 508^37^. As a result, hydration of respiratory surfaces cannot be ensured, leading to cystic fibrosis. Examination of various potentiators and inhibitors are important to study folding and resolve CFTR dysfunction^38^.

The aim of this work was to develop an in vitro PylRS-AF based translation system to gain high aminoacylation- and suppression efficiencies with bulky ncaa. The performance of the aaRS was analyzed in CHO and *Sf*21 cell lysates, since both cell-free systems are most suitable for the production of complex membrane proteins. The complex membrane protein Adora2a was utilized to examine suppression efficiency under various synthesis conditions. The findings were subsequently transferred to the production of the anion channel to demonstrate the applicability to another pharmacologically relevant protein.

## Methods

### Cell-free protein synthesis reactions

The plasmids for CHO and *Sf*21 based cell-free synthesis of PylRS-AF, CFTR, CFTR-ambF157 and CFTR-ambF337 were cloned into a pUC57-1.8K backbone and obtained by de novo gene synthesis (Biocat). A CrPV IRES site is located upstream of the coding sequence to initiate protein translation. The coding sequence of the PylRS-AF contained the mutations Y306A and Y384F as previously reported^16^. The phenylalanine codon at either amino acid position 157 or 337 in the CFTR coding sequence, was substituted by an amber stop codon (CFTR-ambF157 and CFTR-ambF337). CHO and *Sf*21 cell lysates were prepared as previously described^32,39,40^.

Batch based cell-free reactions were composed of 40% CHO or *Sf*21 cell lysate, 10 µM PolyG, 30 mM HEPES–KOH (pH 7.5, Carl Roth GmbH), 100 mM sodium acetate (Merck), 3.9 mM magnesium acetate (Merck), 150 mM potassium acetate (Merck), 100 µM amino acids (Merck), 250 µM spermidin (Roche), 2.5 mM Dithiothreitol (Life technologies GmbH), 100 µg/ml creatine phosphokinase (Roche), 20 mM creatine phosphate (Roche), 1.75 mM ATP (Roche), 0.3 mM GTP (Roche), 0.3 mM of UTP (Roche), 0.3 mM CTP (Roche), 0.1 mM of the cap analogue m7G(ppp)G (Prof. Edward Darzynkiewicz, Warsaw University, Poland) and 1 U/μl T7 RNA polymerase (Agilent). Radioactive ^14^C-leucine (specific radioactivity 46.15 dpm/pmol, Perkin Elmer) was added to the reaction (final concentration 30 µM) to enable qualitative and quantitative analysis of radio-labeled proteins. Batch based reactions were incubated for three hours at 600 rpm. Continuous exchange cell-free (CECF) reactions were carried out as previously described^41^. Briefly, the reaction mix differs from batch based cell-free reactions by the use of 4.5 µM PolyG, 5 µM ^14^C-leucine and the addition of 0.02% sodium azide (Merck) and 30 µM of the caspase inhibitors Ac-DEVD-CMK (Promega) and Z-VAD-FMK (Promega) for CHO and *Sf*21 based reactions, respectively. The feeding mixture was composed of HEPES–KOH (f.c. 30 mM, pH 7.6), 3.9 magnesium acetate, 150 mM potassium acetate, 100 µM amino acids, 250 µM spermidine, 20 mM creatine phosphate, 1.75 mM ATP, 0.3 mM GTP, 0.3 mM of UTP, 0.3 mM CTP, 0.33 mM of the cap analogue m7G(ppp)G, 0.02% sodium azide, 30 µM caspase inhibitor and 5 µM radioactive ^14^C-leucine. Furthermore, CHO based CECF reactions were carried out without Cap analogue and with 18.5 mM creatine phosphate. The plasmid concentration was 60 ng/µl unless otherwise stated. Dialysis devices (Scienova) were filled with reaction mix and feeding mix and incubated at 600 rpm for up to 48 h at 30 °C for CHO based CECF reactions and for 24 h at 27 °C for *Sf*21 based CECF reactions. The plasmids pIVEX-2.4d-PylS-AF and pIVEX-1.3WG-His-PylRS-AF were used to synthesize PylRS-AF in cell-free reactions using the RTS 500 *E.coli* HY Kit (biotechrabbit) and RTS 500 Wheat Germ CECF Kit (biotechrabbit), respectively, according to the manufacturer’s instructions. Due to the presence of ^14^C-leucine during cell-free protein synthesis, the concentration of radiolabeled proteins can be determined quantitatively by liquid scintillation counting after hot trichloroacetic acid precipitation as described previously^41^. The radioactive protein bands in the SDS-PAGE gel were quantified using the ImageQuant TL Software.

### Orthogonal cell-free reactions

Orthogonal cell-free reactions were further supplemented with 2 µM orthogonal tRNAPyl(CUA) and 2 mM strained cyclooctyne (SCO). In the case of CECF reactions, 2 mM of SCO was also added to the feeding mix. The PylRS-AF was either added to the batch reaction in the form of supernatant of another cell-free reaction or supplemented in the form of plasmid DNA. The plasmids A2aRamb and A2aR for cell-free synthesis of adenosine A2a receptor (with and without an amber stop codon at amino acid position 215) were utilized as previously described^33^. A Nanoluciferase sequence was linked to the C-terminus of the receptor to evaluate amber stop codon suppression in the presence or absence of the orthogonal components. The plasmid of PylRS-AF was added to the orthogonal cell-free reaction containing A2aRamb at different time points and amber stop codon suppression was analyzed based on the Nano-Glo Luciferase Assay System (Promega). The luciferase assay was performed in technical duplicates.

### Fluorescent labeling

The suspension of orthogonal cell-free reactions was centrifuged at 16,000 × g for 10 min at 4 °C and the pellet was resuspended in the same volume PBS. A click reaction with 5 µM fluorescent dye Tetrazine-Cy5 (Jena Bioscience) and 5 µl solubilized pellet, which contained proteins with incorporated SCO was filled up to 10 µl with PBS and incubated for 10 min at 25 °C and 500 rpm. Afterwards proteins were precipitated by acetone.

### Planar Bilayer Electrophysiology

The translation mixture of cell-free produced CFTR variants was centrifuged for 10 min at 16,000 × g at 4 °C and the pellet was resuspended in an equal volume PBS. The resuspended pellet contained the microsomal vesicles and was utilized for electrophysiological measurements. Planar bilayer experiments were performed as explained previously^42,43^. Lipid bilayers were formed from 1, 2‐diphytanoyl‐sn‐glycero‐3‐phosphocholine (DPhPC) (Avanti Polar Lipids, Albaster, AL, USA). Lipids were dissolved in octane (Sigma Aldrich, Munich, Germany) at a concentration of 10 mg/ml. 10 mM HEPES, 150 mM NaCl [Sigma Aldrich (Fluka), Munich, Germany] pH 7.0 was used as an electrolyte. 5 µl of the ER derived vesicles suspended in PBS were added to the chamber containing the buffer and waited until the visible response^44^. For current measurements, different voltages were applied to analyze the functional properties. For blockage studies, 1 mM GlyH101 was added to the buffer solution while recording. The cavity contains the non-polarizable working electrode containing Ag/AgCl layer deposited on the underlying Cr/Au layer. Briefly, 180 µl of electrolyte solution was added to the measurement chamber of an Orbit 16 System (Nanion Technologies GmbH, Munich, Germany). A single channel amplifier (EPC-10, HEKA Electronic Dr. Schulze GmbH, Lambrecht, Germany) was connected to the multiplexer electronics port of the Orbit16 system. Recordings were done at a sampling rate of 50 kHz with a 10 kHz Bessel filter. Data were analysed with Clampfit (Molecular Devices, Sunnyvale, CA, USA).

## Results

The specific feature of the PylRS-AF double mutant to accept bulky amino acids has prompted us to optimize synthesis conditions for site-specific labeling of complex membrane proteins in cell-free protein synthesis reactions. However, PylRS was found to be mainly abundant in the insoluble fraction in *E.coli* cell based production^45^. Jiang and Krzycki reported that PylRS is prone to aggregation due to the instable N-terminal domain, thus hampering amber suppression^46^. Therefore, our attempt was to generate a production environment, which does not require protein purification, thereby minimizing protein instability or aggregation.

### Comparison of cell-free synthesis platforms

**Figure 2:**
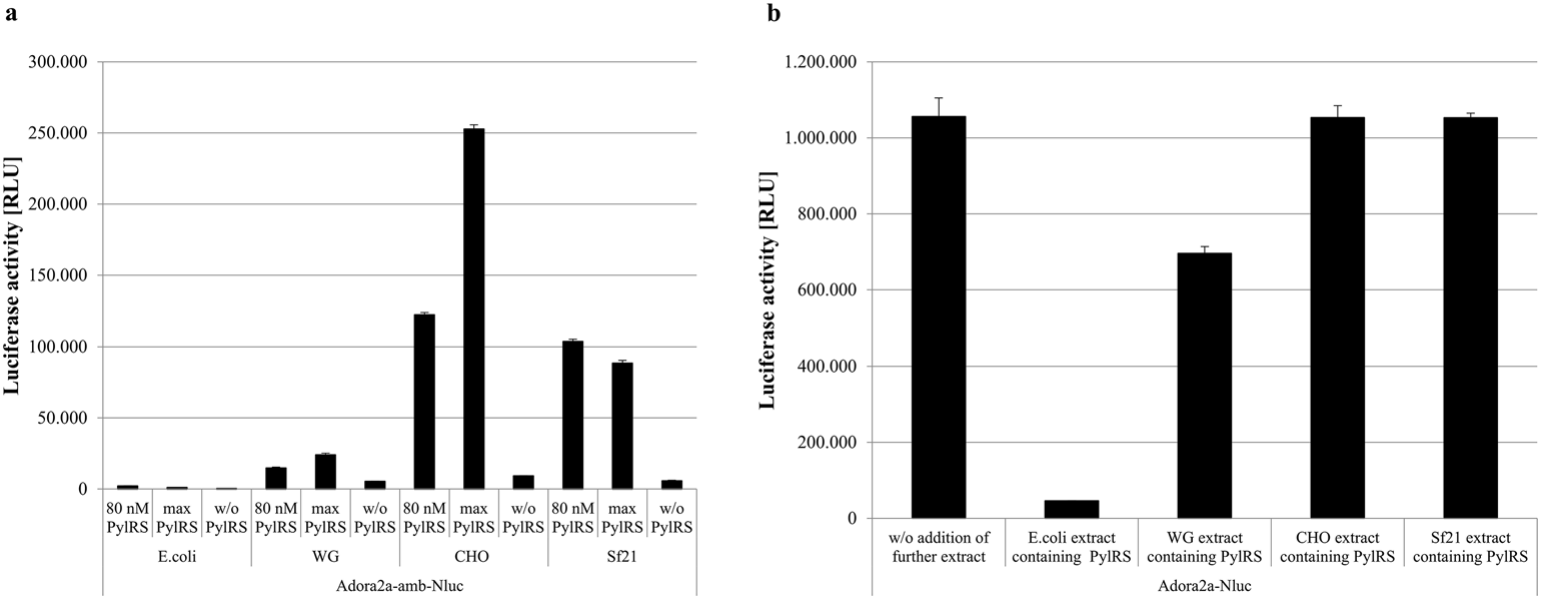
Detection of amber-suppression efficiency in cell-free protein synthesis. (**a**) PylRS-AF was synthesized in cell-free reactions based on *E.coli*, wheat germ (WG), *Sf*21 and CHO in a CECF format. The supernatant of these reactions was supplemented to a CHO batch based cell-free reaction containing Adora2a-amb-Nluc as template. In presence of active PylRS the full length protein with C-terminal luciferase was obtained. Nluc activity was measured after three hours synthesis reaction. The maximal possible PylRS-AF concentration and equal enzyme concentrations (80 nM) were added to the batch based reaction. (**b**) The same supernatant described in (**a**) was supplemented to a CHO based cell-free reaction containing Adora2a-Nluc to determine the effect of supernatants on the translation reaction. Nluc activity was measured after three hours synthesis reaction. Measurements were performed in duplicate. Data are shown as mean ± SD. Independent experiments were performed two times and can be found in the Suppl. Fig. S11.

Identification of optimal translation conditions for active PylRS-AF is required to gain a highly productive system, while retain catalytic activity. Considering the reported instability of the enzyme in vivo, cell-free synthesis can be a powerful alternative to modify reaction conditions. Usually, purified orthogonal components are supplemented to the cell-free reaction as previously shown with a mutant tyrosyl-tRNA synthetase^47^. A two-step reaction was used to overcome purification steps, which may interfere with enzyme stability of the PylRS-AF. In a first reaction, enzyme is produced and the supernatant is collected. Afterwards, the supernatant containing the PylRS-AF is transferred to a second batch based reaction comprising compounds that are crucial for the orthogonal translation reaction, including tRNA(Pyl), the gene of interest with an amber stop codon at a desired position and the ncaa SCO. To test the functionality of this hypothesis, an insect and CHO based cell-free platform and two commercially available cell-free systems based on *E.coli* and wheat germ (WG) were utilized to synthesize PylRS-AF in a continuous-exchange cell-free (CECF) format, wherein fresh substrates and inhibitory by-products are exchanged by a semi-permeable membrane. Afterwards, a batch synthesis with CHO and *Sf*21 cell lysates was chosen to analyze amber suppression efficiency of newly synthesized aaRS, since both systems enable the production of complex proteins, such as membrane proteins. Previously, we have shown that in the absence of orthogonal tRNA and SCO amber stop codon readthrough is minimal^48^. As a control reaction, PylRS-AF was omitted in the cell-free reaction. The maximal possible PylRS-AF concentration in a batch based reaction and equal enzyme concentrations (80 nM) were applied to identify the most efficient cell-free production system. The incorporation efficiency was estimated by a reporter gene assay based on Adora2a coupled with a Nano-luciferase (Nluc). Suppression product will be produced if the amber stop codon at amino acid position 215 of the Adenosine receptor 2a (Adora2a-amb-Nluc) would be addressed by the amino-acylated tRNA(Pyl). Consequently C-terminal Nluc will be produced and Nluc activity can be measured (Fig. 1). Concurrently, the release factor outcompetes the orthogonal translation system by terminating protein translation resulting in termination product without Nluc activity. It is noticeable that CHO and *Sf*21 PylRS-AF produced highest Nluc activity (Fig. 2a). In contrast, PylRS-AF produced in *E.coli* and WG lysates resulted in up to 121-fold lower luminescence signals in the CHO cell-free synthesis reaction. However, it has to be emphasized that the transferred supernatant may contain inhibitory products affecting general protein synthesis. Therefore, a control CHO based reaction supplemented with cell lysate of different origins and Adora2a-Nluc without an amber codon position was included (Fig. 2b). A 1.5 fold inhibiting effect of WG PylRS-AF was observed compared to Adora2a-Nluc without further supplements. Although, *E.coli* cell lysate drastically reduced protein synthesis and luminescence units decreased by 23-fold, it is not surprising that a prokaryotic system can interfere with eukaryotic transcription and translation. Nonetheless, amber suppression could be increased up to 24% by adding the largest possible quantity of CHO PylRS-AF, while supplementation of 80 nM PylRS-AF gained suppression efficiencies of 11.6% (CHO), 9.9% (*Sf*21), 4.5% (*E.coli*) and 2.1% (WG).

The same approach was transferred to a *Sf*21 batch based reaction, corroborating CHO and *Sf*21 produced PylRS-AF as the most active ones (Suppl. Fig. S1). *Sf*21 and CHO lysates were utilized for further optimization steps as they were the most compatible for the targeted approach and produced active PylRS-AF.

**Figure 3:**
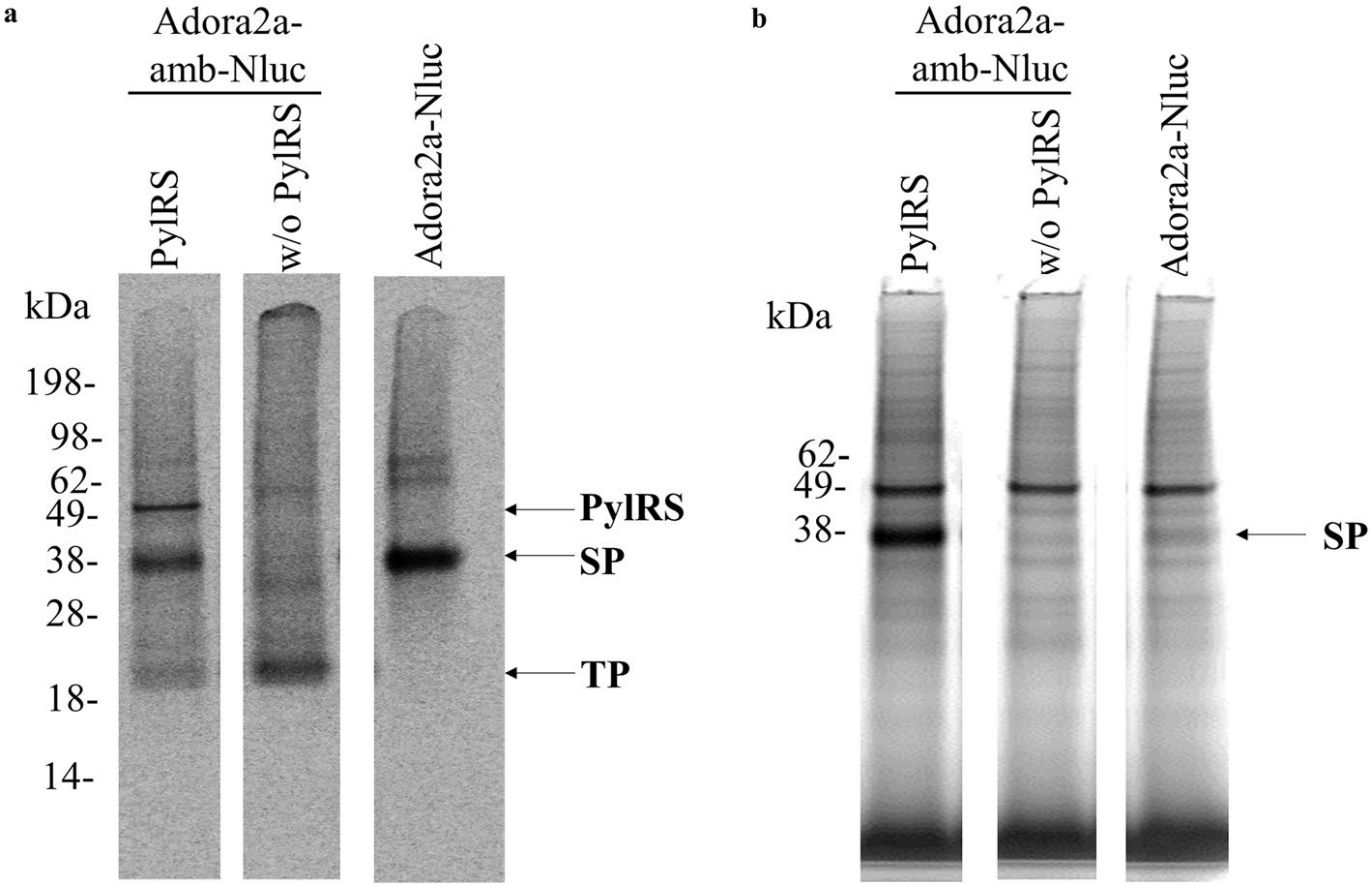
Site-specific incorporation of SCO into Adora2a in *Sf*21 lysate. The non-canonical amino acid SCO was incorporated into Adora2a-amb-Nluc during a *Sf*21 batch based cell-free reaction in the presence or absence of PylRS-AF. Afterwards the modified Adora2a was coupled to a tetrazine Cy5 dye and visualized by excitation utilizing a 633 nm laser. (**a**) Autoradiography showing the full length protein in the presence of PylRS, the termination product in the absence of PylRS and the full length protein without amber stop codon. (**b**) In-gel fluorescence showing an intense fluorescent band only in the presence of the Nluc template harbouring an amber stop codon and in the presence of PylRS. SP: Suppression product. TP: Termination product. Uncropped autoradiography images are included in Supplementary information.

The Adora2a-amb-Nluc construct was further utilized to visualize in-gel fluorescence by tetrazine ligation with a fluorescent tetrazine Cy5 dye. Therefore, the CHO-PylRS-AF crude supernatant mixture was transferred to a *Sf*21 batch based cell-free reaction. Intensive protein bands could be displayed by autoradiography (Fig. 3a) as well by fluorescence (Fig. 3b). The suppression efficiency estimated by protein bands was approximately 23%, which was in accordance with observations by the Nluc-assay (Supplementary Fig. S9).

### Co-expression approach

**Figure 4:**
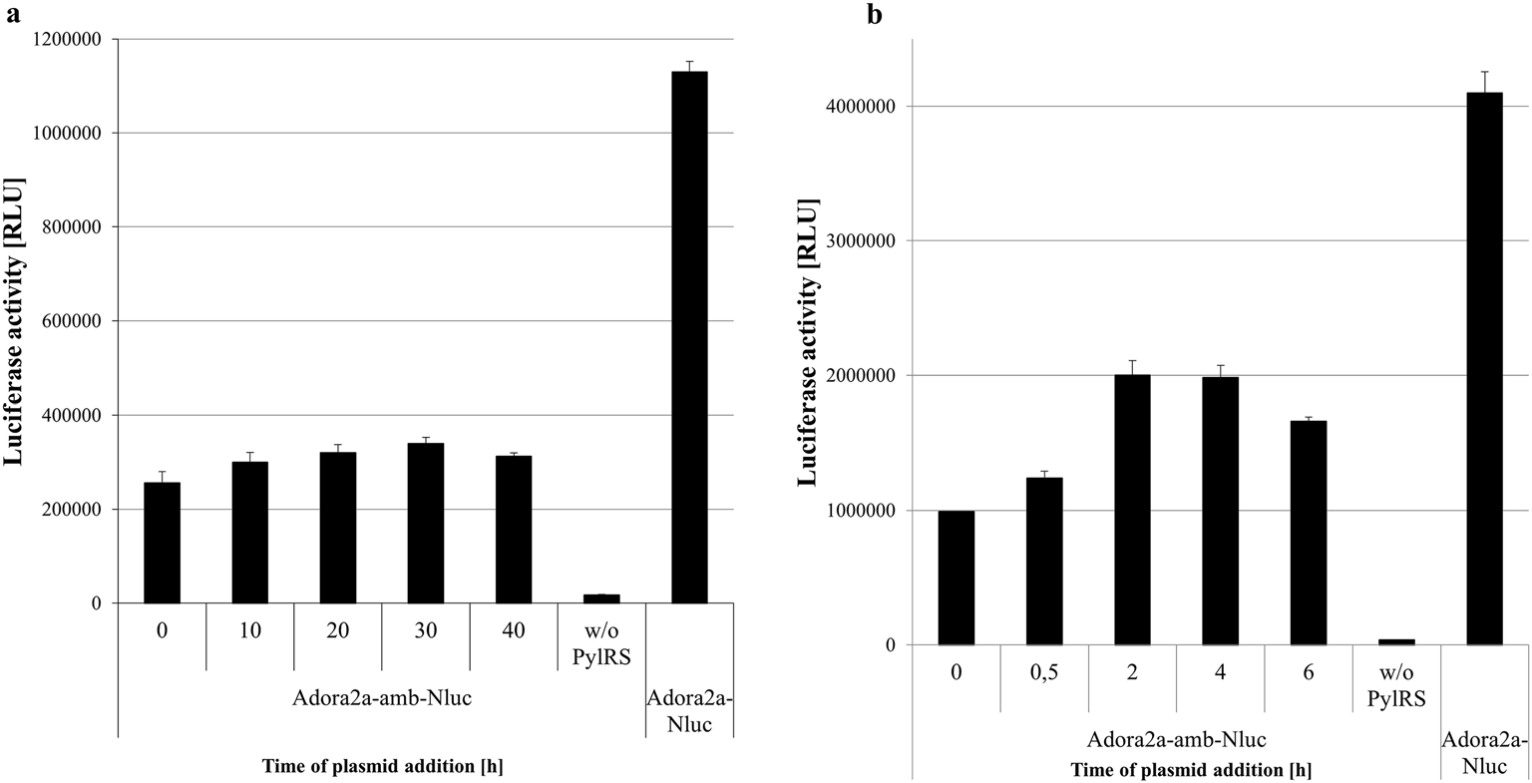
Luciferase activity of co-expressed plasmids in *Sf*21 cell-free systems. PylRS-AF was co-expressed with Adora2a-amb-Nluc. The PylRS template was directly added at the beginning of the reaction. The Adora2a-amb-Nluc template was added after the indicated time. (**a**) Batch based reaction showing the successful synthesis of full length protein in the presence of PylRS. The addition of the Adora2a-amb-Nluc template after 30 min resulted in the highest suppression efficiency. (**b**) CECF-reaction reaction showing the successful synthesis of full length protein in the presence of PylRS. The addition of the Adora2a-amb-Nluc template between 2 and 4 h resulted in the highest suppression efficiency. Measurements were performed in duplicate. Data are shown as mean ± SD.

In order to simplify the orthogonal translation with the PylRS-AF/tRNA(Pyl) system, templates of the aaRS and Adora2a-amb-Nluc were co-expressed in one cell-free reaction. In this experiment, the enzyme can aminoacylate SCO-tRNA(Pyl) immediately after its synthesis, without disturbing the catalytic activity. By varying the time points for addition of Adora2a-amb-Nluc plasmid an incorporation efficiency of 30% in batch based and up to 49% in CECF reactions could be achieved in *Sf*21 cell lysate (Fig. 4). Early addition of Adora2a-amb-Nluc caused reduced luminescence signals, due to a low amount of PylRS-AF resulting in competition of simultaneously transcription of both templates. During continuous reaction, a higher PylRS-AF concentration promotes amber suppression by increasing aminoacylation events. For site-specific incorporation of SCO into the Adora2a-amb-Nluc an incubation time of 30 min in batch based and 2–4 h in CECF based reactions resulted in the highest suppression efficiency. Late supplementation of Adora2a-amb-Nluc plasmid caused diminished amber suppression, considering substrate supply and energy consumption during ongoing cell-free reactions. The intensity of suppression products and the truncated protein in the autoradiogram correlated with results obtained by the NLuc-assay (Suppl. Fig. S2). The same approach was applied to CHO cell-free reactions to test whether this system is also susceptible for co-expression. Similar findings, but with a reduced incorporation efficiency, could be observed (Suppl. Fig. S3).

### Site-specific incorporation of SCO into CFTR

The opportunity to introduce fluorescence labels at defined positions provides a major advantage for analyzing dynamics and folding of proteins such as CFTR. Therefore, the combination of the modification system based on the PylRS-AF/tRNA(Pyl) pair and cell-free synthesis provides high-speed click reactions with high-throughput.

**Figure 5:**
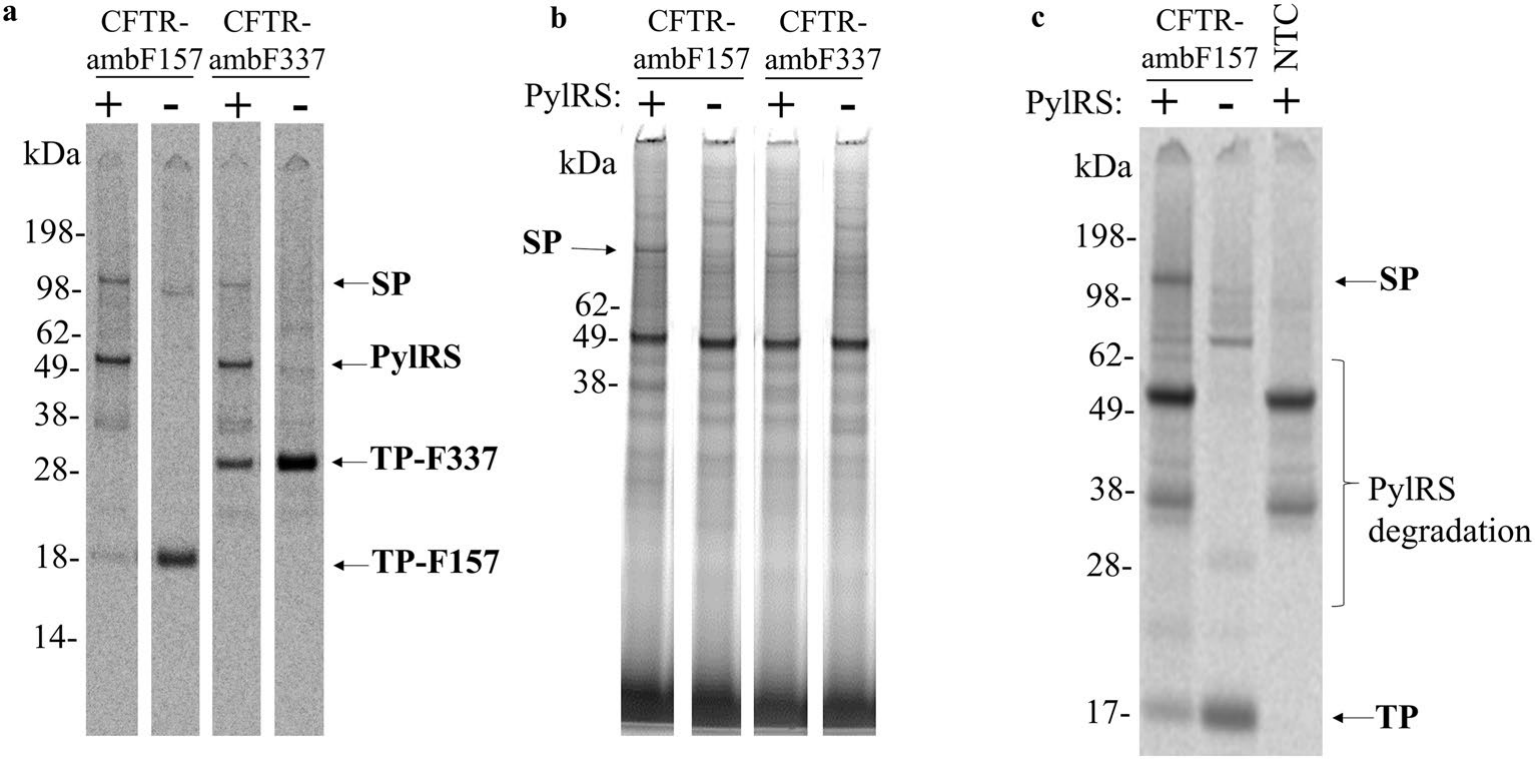
Site-specific incorporation of SCO into CFTR. The non-canonical amino acid SCO was incorporated into CFTR-ambF157 and –F337 during a *Sf*21 batch and CECF based cell-free reaction in the presence or absence of PylRS-AF. Afterwards the modified CFTR variants were coupled to a tetrazine Cy5 dye and visualized by excitation utilizing a 633 nm laser. (**a**) Autoradiography of a batch based *Sf*21 cell-free reaction showing the full length proteins in the presence of PylRS and the specific termination products in absence of PylRS. (**b**) In-gel fluorescence corresponding to the autoradiograph shown in (**a**), which shows a fluorescent band at the marked molecular weight (SP) only in the presence of the CFTR template harbouring an amber stop codon and in presence of PylRS. (**c**) Autoradiography of a CECF based *Sf*21 cell-free reaction showing the full length proteins in the presence of PylRS and the specific termination products in the absence of PylRS, as well as protein bands corresponding to PylRS degradation. SP: Suppression product. TP: Termination product. Uncropped autoradiography images are included in Supplementary information.

Again, supernatant of PylRS-AF was utilized to site-specifically introduce SCO into CFTR at two defined positions and subsequently “click” the suppression product with tetrazine-Cy5 dye. One amber stop codon position was introduced at F157, which is located at the extra-microsomal side of the ion channel. The second position F337 is located in the lumen of the ER-derived microsomes. Amber suppression of both CFTR-amb-constructs could be successfully demonstrated by autoradiography (Fig. 5a). However, amber suppression of CFTR-ambF337 was reduced compared to CFTR-ambF157. Moreover, fluorescent protein bands could not be determined for F337 (Fig. 5b). This may be attributed to the luminal location of F337, whereby the fluorescent dye is hindered from crossing the lipid layer. However, the low amount of F337 suppression product had also a large impact on the overall yield of fluorescent CFTR. Nevertheless, successful ligation of CFTR-ambF157-SCO to the fluorescent tetrazine dye was visualized. Therefore, the CFTR-ambF157 construct was utilized for the production of amber-suppressed CFTR in a CECF-format. Indeed, a suppression efficiency of ~ 61% could be achieved by transferring the co-expression approach from GPCR cell-free orthogonal synthesis with PylRS-AF to orthogonal cell-free CFTR production (Fig. 5c, Supplementary Fig. S10).

### Functional analysis of amber suppressed CFTR-ambF157

**Figure 6:**
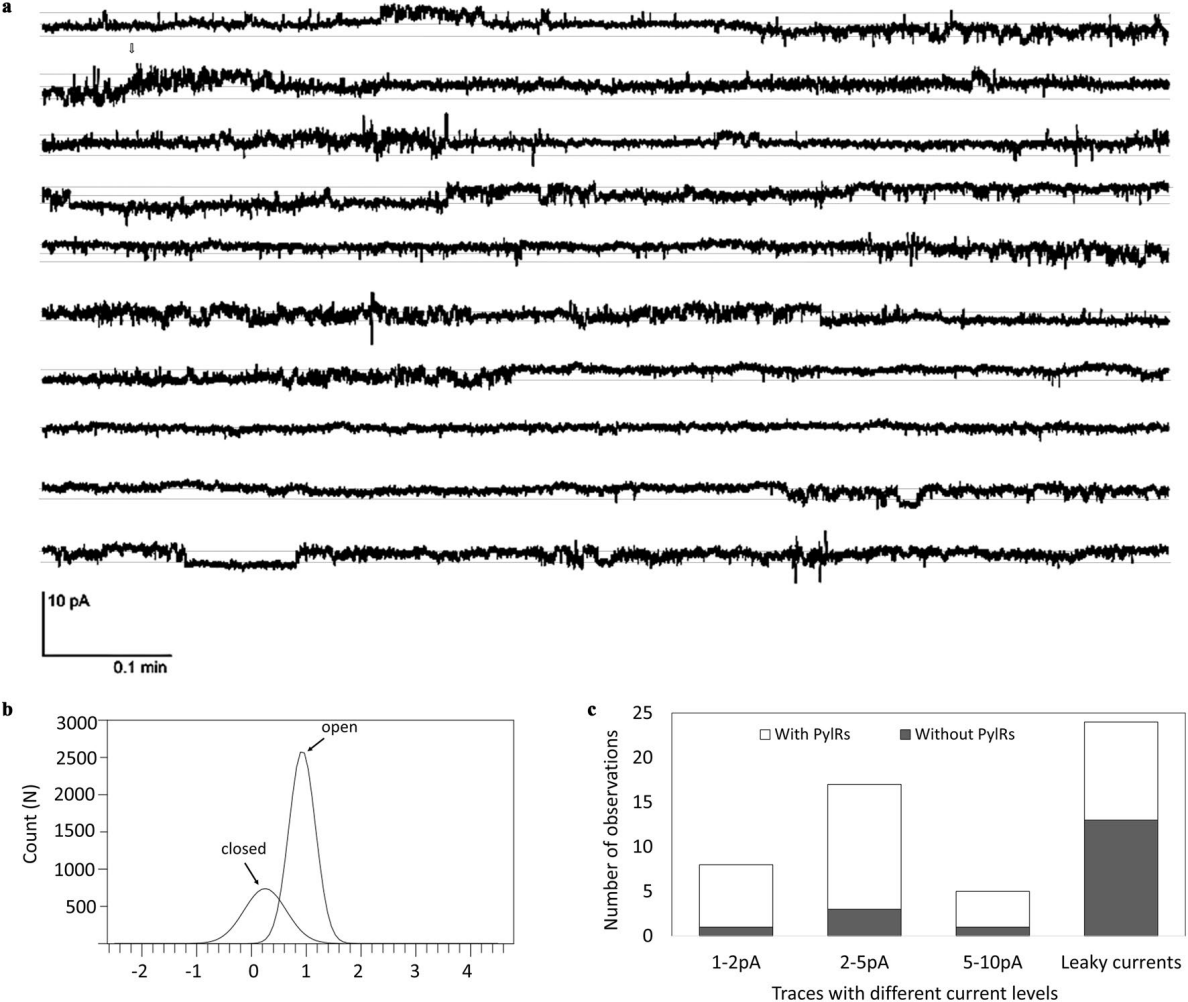
Planar bilayer measurements of the CFTR-ambF157 with PylRS-AF channel function. (**a**) Single-channel activity recordings from CFTR-ambF157 with PylRS-AF incorporated microsomes reconstituted into planar DPhPC lipid bilayers at + 60 mV. (**b**) All point histogram of the corresponding trace showing two levels corresponding to open (1 pA) and closed (0 pA) transient states of the ion channel. (**c**) Comparison of different levels of currents recorded from planar bilayers for CFTR-ambF157 with (white) and without (grey) PylRS-AF while recording the single-channel activity. All recordings were made under symmetrical 10 mM HEPES, 150 mM NaCl, pH 7.0, buffer solutions in the presence of 8–12 mM ATP and 30–62.5 U/ml PKA (n = 6).

The next step was to determine whether modified CFTR was able to retain its functionality. For this purpose, single channel activity was measured from the lipid bilayers after the addition of microsomes harboring the CFTR-ambF157 (both with and without PylRS-AF during cell-free synthesis). All planar lipid bilayer measurements were done in the presence of 10 mM HEPES, 150 mM NaCl, pH 7.0. All measurements were performed in the presence of 8–12 mM ATP and 30–62.5 U/ml PKA starting with 8 mM ATP and 30 U/ml PKA and adding extra ATP and PKA in the middle so that final concentrations reached the above mentioned maximal concentrations. Once the samples are injected, preincubation was done at least for 10 min to make sure the samples fuse to the lipid bilayer. Control measurements were done without the sample, but with ATP and PKA over the planar lipid bilayer to measure the impact of the compounds on the lipid bilayer. A 5 min single channel trace of CFTR-ambF157 with incorporated SCO from vesicle reconstituted lipid bilayer is shown in Fig. 6a with clear closing and opening transition levels at + 60 mV. The currents are comparatively larger (1–2 pA) in reference to the literature values, which might be due to the currents from multichannel openings as bursts with some sub-conductance levels corresponding to the single channel insertions^49,50^. However, channel recordings are inconsistent with rapid flickering activity with currents jumping between open and closed transitions and even to multi-conductance states. All point histograms were plotted from the recordings of the Fig. 6a which showed two clearly defined levels corresponding to closing and open transition states (Fig. 6b). There was always an overlap between the transition levels, which indicates there is a lot of flickering during the recordings. This could be due to the interference from the cell-free components as the activity was directly measured from the protein incorporated in microsomes without any purification. Another reason could be due to the influence of HEPES component in the buffer over the gating pattern and small conformational shifts that might be responsible for partial closing or opening of the channel^51^. As the currents were inconsistent with multiple insertions, it was challenging to measure the stable single channel recordings and often the channels open in clusters to different levels. The channel was fluctuating between multiple levels (ranging from 1–5 pA). We segregated the activity based on current levels for both CFTR-ambF157 with and without PylRS-AF. Currents within the range of 1–2 pA were due to the single channel activity and currents of 2–5 pA were assumed to be due to multi-channel activity. Even higher levels of 5–10 pA also contributed to large activity attributing to multichannel conductance (Fig. 6c). This multichannel conductance might be due to the rapid insertions of channels into the planar lipid bilayer and due to the presence of excess ATP and PKA during the process of the experiment, which might increase the activity. There were often irregular leaky currents due to the tension created by the microsomes onto the planar bilayer. These currents were difficult to control and often leading to planar bilayer rupture^50^. These were observed regularly in both cases. According to the plots, in the case of CFTR-ambF157 with PylRS-AF, there is more than 80% of activity compared to the bilayers recorded from the CFTR-ambF157 without PylRS-AF samples (total 6 experiments with more than 70 bilayer recordings). While channel conductance of full-length CFTR was expected, channel activity of CFTR-ambF157 without PylRS-AF could be due to readthrough of the amber stop codon and thus synthesis of full-length protein in the absence of PylRS-AF. However, based on autoradiography and fluorescence analysis, no full-length CFTR was observed in the absence of PylRS-AF. Currents with stable recordings with similar gating pattern from CFTR-ambF157 with PylRS-AF are depicted in Suppl. Figs. S4–S6.

In the Suppl. Figs. S4 and S6, single channel recordings from multiple experiments were plotted with their corresponding histograms. All recordings from these experiments showed the similar behaviour as presented in Fig. 6. Blocking experiments were conducted in the presence of CFTR specific blocker GlyH-101 (Suppl. Fig. S5). Initially the currents were recorded at + 60 mV from the CFTR-ambF157 with PylRS-AF samples and after some time 3 µl of 1 mM GlyH-101 was added to the planar bilayer. We noticed that rapid activity was decreased and after additional recording, 2 µl of blocker was added additionally. The activity was completely blocked at a concentration of 25 µM GlyH-101. Often addition of blockers even lead to disruption of the bilayers.

## Discussion

The present findings demonstrate that purification of orthogonal aaRS can be circumvented by adding the enzyme as a supernatant to the open cell-free system or co-expressing it during target protein synthesis. Thus, adenosine receptor A2a could be modified by incorporating SCO in cell-free systems with a suppression efficiency of up to 49% using established methods. In contrast, Meineke et al. achieved ~ 50% amber suppression by incorporating exo-bicyclo-nonyne–L-lysine (BCN) and trans-cyclooct-2-ene–L-lysine (TCO) with PylRS-AF in vivo^52^. On the other hand, suppression efficiencies did not exceed 4% by introducing SCO, BCN and TCO by a range of PylRS variants including the double mutant PylRS-AF in *E.coli* cell-free systems^53^. However, it has to be emphasized that different expression systems, proteins and amber stop codon positions were used. The level of amber suppression depends on several factors, including the local mRNA sequence context. Pott and co-workers identified individual sequence preferences on the mRNA transcript in *E.coli* for various orthogonal aaRS like *M. mazei* PylRS^54^. Indeed, the sequence preference in eukaryotic systems may be different and many efforts have to be done to elucidate the combination of sequence context and additional factors of the translational apparatus^55^.

Nonetheless, site-specific incorporation of SCO into the Adenosine receptor A2a could be empowered with the co-expression approach by implementing an environment without perturbing enzyme stability. Moreover, the approach is rapid and addition of PylRS-AF template is cost-effective compared to synthesizing the enzyme separately. Observations are consistent with a recently published study in a release factor 1 depleted *E.coli* cell-free system^56^. Co-expression of bovine fatty acid binding protein (FABP) and PylRS-AF with either SCO or BCN was utilized to couple FABP with fluorescent tetrazine-Cy3. Alternatively, Beránek et al. examined an evolved *Methanomethylophilus alvus* PylRS lacking the N-terminal domain, which is required for *M. mazei* PylRS-AF activity to overcome the instable nature of full-length PylRS^57^. Indeed, *M. alvus* PylRS was engineered to accept ncaa such as Nε-[(tert-butoxy)carbonyl]-L-lysine, which is a known substrate of *M. mazei* PylRS-AF, but it is not clear if the unique substrate specificity of the AF-double mutant can be transplanted to the *M. alvus* PylRS in order to incorporate large and bulky ncaa.

Previous work demonstrated that orthogonal aaRS can be transiently and stably transfected into CHO cells and subsequent cell lysate containing active *E. coli* tyrrosyl tRNA synthetase and PylRS-AF were used for cell-free protein synthesis^48^. While this approach also bypasses the purification of orthogonal aaRS, the time required is significantly increased. This constrains the advantages of cell-free systems to rapidly utilize diverse orthogonal systems for the modification of difficult-to-express proteins.

The present work successfully demonstrates that both cell-free systems, *Sf*21 and CHO, which are also often used as cell-based protein production systems, represent a promising route for the production of site-specifically labeled complex proteins. Hence, CFTR has been used to produce another pharmacologically important ion channel in an active and modified form. The examination of CFTR folding and function with diverse activators and inhibitors is required for the treatment of cystic fibrosis^38^. One promising approach to determine folding events is fluorescence energy transfer between a donor and acceptor molecule^58^. Khushoo et al. revealed incorrect folding of the first CFTR nucleotide-binding domain in a co-translational process by incorporating an N-terminal cyan fluorescent fusion protein and a small fluorophore at one engineered amber stop codon position^59^. Utilization of this fluorescence resonance energy transfer based approach ensures flexible incorporation of fluorophores at specific positions, thus highlighting the significance of protein modification by amber suppression. Therefore, the developed system provides a good starting point to assay CFTR variants, which are modified at desired sites and investigate its functionality. Moreover, a recent study comprehensively demonstrated the potential of cell-free protein synthesis by producing diverse ion channels based on wheat germ lysates^60^. In combination with orthogonal systems, a variety of pharmacologically relevant targets can be now modified and efficiently evaluated in a small scale.

### Supplementary Information

Supplementary Figures.

## Data Availability

All data generated or analyzed during this study are included in this published article (and its Supplementary Information files).

## References

[1] Snapp E (2005). Design and use of fluorescent fusion proteins in cell biology. Curr. Protoc. Cell Biol. Chapter.

[2] Proft T (2010). Sortase-mediated protein ligation: An emerging biotechnology tool for protein modification and immobilisation. Biotechnol. Lett..

[3] Mills KV, Johnson MA, Perler FB (2014). Protein splicing: How inteins escape from precursor proteins. J. Biol. Chem..

[4] Berrade L, Camarero JA (2009). Expressed protein ligation: A resourceful tool to study protein structure and function. Cell. Mol. Life Sci..

[5] Day RN, Davidson MW (2009). The fluorescent protein palette: Tools for cellular imaging. Chem. Soc. Rev..

[6] Debelouchina GT, Muir TW (2017). A molecular engineering toolbox for the structural biologist. Q. Rev. Biophys..

[7] deGruyter JN, Malins LR, Baran PS (2017). Residue-specific peptide modification: A chemist's guide. Biochemistry.

[8] Hao Z, Hong S, Chen X, Chen PR (2011). Introducing bioorthogonal functionalities into proteins in living cells. Acc. Chem. Res..

[9] Quast RB, Mrusek D, Hoffmeister C, Sonnabend A, Kubick S (2015). Cotranslational incorporation of non-standard amino acids using cell-free protein synthesis. FEBS Lett..

[10] Arranz-Gibert P, Patel JR, Isaacs FJ (2019). The role of orthogonality in genetic code expansion. Life.

[11] Bianco A, Townsley FM, Greiss S, Lang K, Chin JW (2012). Expanding the genetic code of Drosophila melanogaster. Nat. Chem. Biol..

[12] Wan W, Tharp JM, Liu WR (2014). Pyrrolysyl-tRNA synthetase: An ordinary enzyme but an outstanding genetic code expansion tool. Biochim. Biophys. Acta.

[13] Tian Y, Jacinto MP, Zeng Y, Yu Z, Qu J, Liu WR, Lin Q (2017). Genetically encoded 2-Aryl-5-carboxytetrazoles for site-selective protein photo-cross-linking. J. Am. Chem. Soc..

[14] Schmidt MJ, Borbas J, Drescher M, Summerer D (2014). A genetically encoded spin label for electron paramagnetic resonance distance measurements. J. Am. Chem. Soc..

[15] Plass T, Milles S, Koehler C, Schultz C, Lemke EA (2011). Genetically encoded copper-free click chemistry. Angew. Chem. Int. Ed. Engl..

[16] Yanagisawa T, Ishii R, Fukunaga R, Kobayashi T, Sakamoto K, Yokoyama S (2008). Multistep engineering of pyrrolysyl-tRNA synthetase to genetically encode N(epsilon)-(o-azidobenzyloxycarbonyl) lysine for site-specific protein modification. Chem. Biol..

[17] Kurra Y, Odoi KA, Lee Y-J, Yang Y, Lu T, Wheeler SE, Torres-Kolbus J, Deiters A, Liu WR (2014). Two rapid catalyst-free click reactions for in vivo protein labeling of genetically encoded strained alkene/alkyne functionalities. Bioconjug. Chem..

[18] Oliveira BL, Guo Z, Bernardes GJL (2017). Inverse electron demand Diels-Alder reactions in chemical biology. Chem. Soc. Rev..

[19] Kozma E, Demeter O, Kele P (2017). Bio-orthogonal fluorescent labelling of biopolymers through inverse-electron-demand Diels-alder reactions. ChemBioChem.

[20] Li L, Zhang Z (2016). Development and applications of the copper-catalyzed azide-alkyne cycloaddition (CuAAC) as a bioorthogonal reaction. Molecules.

[21] Lu Y (2017). Cell-free synthetic biology: Engineering in an open world. Synth. Syst. Biotechnol..

[22] Takimoto JK, Xiang Z, Kang J-Y, Wang L (2010). Esterification of an unnatural amino acid structurally deviating from canonical amino acids promotes its uptake and incorporation into proteins in mammalian cells. ChemBioChem.

[23] Lin X, Yu ACS, Chan TF (2017). Efforts and challenges in engineering the genetic code. Life.

[24] Stech M, Nikolaeva O, Thoring L, Stöcklein WFM, Wüstenhagen DA, Hust M, Dübel S, Kubick S (2017). Cell-free synthesis of functional antibodies using a coupled in vitro transcription-translation system based on CHO cell lysates. Sci. Rep..

[25] Zemella A, Thoring L, Hoffmeister C, Kubick S (2015). Cell-free protein synthesis: pros and cons of prokaryotic and eukaryotic systems. ChemBioChem.

[26] Adachi J, Katsura K, Seki E, Takemoto C, Shirouzu M, Terada T, Mukai T, Sakamoto K, Yokoyama S (2019). Cell-free protein synthesis using S30 extracts from Escherichia coli RFzero strains for efficient incorporation of non-natural amino acids into proteins. Int. J. Mol. Sci..

[27] Seki E, Yanagisawa T, Yokoyama S (2018). Cell-free protein synthesis for multiple site-specific incorporation of noncanonical amino acids using cell extracts from RF-1 deletion E. coli strains. Methods Mol. Biol..

[28] Polycarpo CR, Herring S, Bérubé A, Wood JL, Söll D, Ambrogelly A (2006). Pyrrolysine analogues as substrates for pyrrolysyl-tRNA synthetase. FEBS Lett..

[29] Tate CG (2001). Overexpression of mammalian integral membrane proteins for structural studies. FEBS Lett..

[30] Sachse R, Wüstenhagen D, Šamalíková M, Gerrits M, Bier FF, Kubick S (2013). Synthesis of membrane proteins in eukaryotic cell-free systems. Eng. Life Sci..

[31] Fenz SF, Sachse R, Schmidt T, Kubick S (2014). Cell-free synthesis of membrane proteins: Tailored cell models out of microsomes. Biochim. Biophys. Acta.

[32] Brödel AK, Sonnabend A, Kubick S (2014). Cell-free protein expression based on extracts from CHO cells. Biotechnol. Bioeng..

[33] Zemella A, Richter T, Thoring L, Kubick S, Tiberi M (2019). A combined cell-free protein synthesis and fluorescence-based approach to investigate GPCR binding properties. G protein-coupled receptor signaling. Methods and protocols.

[34] Brödel AK, Kubick S (2014). Developing cell-free protein synthesis systems: A focus on mammalian cells. Pharm. Bioprocess..

[35] Saint-Criq V, Gray MA (2017). Role of CFTR in epithelial physiology. Cell. Mol. Life Sci..

[36] Patrick AE, Thomas PJ (2012). Development of CFTR structure. Front. Pharmacol..

[37] Riordan JR (2008). CFTR function and prospects for therapy. Annu. Rev. Biochem..

[38] Thiagarajah JR, Verkman AS (2003). CFTR pharmacology and its role in intestinal fluid secretion. Curr. Opin. Pharmacol..

[39] Thoring L, Wüstenhagen DA, Borowiak M, Stech M, Sonnabend A, Kubick S (2016). Cell-free systems based on CHO cell lysates: optimization strategies, synthesis of "difficult-to-express" proteins and future perspectives. PLoS ONE.

[40] Kubick S, Gerrits M, Merk H, Stiege W, Erdmann VA, DeLucas L, DeLucas LJ (2009). Chapter in vitro synthesis of posttranslationally modified membrane proteins. Membrane protein crystallization.

[41] Thoring L, Dondapati SK, Stech M, Wüstenhagen DA, Kubick S (2017). High-yield production of "difficult-to-express" proteins in a continuous exchange cell-free system based on CHO cell lysates. Sci. Rep..

[42] del Rio Martinez JM, Zaitseva E, Petersen S, Baaken G, Behrends JC (2015). Automated formation of lipid membrane microarrays for ionic single-molecule sensing with protein nanopores. Small.

[43] Baaken G, Ankri N, Schuler A-K, Rühe J, Behrends JC (2011). Nanopore-based single-molecule mass spectrometry on a lipid membrane microarray. ACS Nano.

[44] Dondapati SK, Kreir M, Quast RB, Wüstenhagen DA, Brüggemann A, Fertig N, Kubick S (2014). Membrane assembly of the functional KcsA potassium channel in a vesicle-based eukaryotic cell-free translation system. Biosens. Bioelectron..

[45] Chatterjee A, Sun SB, Furman JL, Xiao H, Schultz PG (2013). A versatile platform for single- and multiple-unnatural amino acid mutagenesis in Escherichia coli. Biochemistry.

[46] Jiang R, Krzycki JA (2012). PylSn and the homologous N-terminal domain of pyrrolysyl-tRNA synthetase bind the tRNA that is essential for the genetic encoding of pyrrolysine. J. Biol. Chem..

[47] Zemella A, Thoring L, Hoffmeister C, Šamalíková M, Ehren P, Wüstenhagen DA, Kubick S (2018). Cell-free protein synthesis as a novel tool for directed glycoengineering of active erythropoietin. Sci. Rep..

[48] Schloßhauer JL, Cavak N, Zemella A, Thoring L, Kubick S (2022). Cell engineering and cultivation of Chinese hamster ovary cells for the development of orthogonal eukaryotic cell-free translation systems. Front. Mol. Biosci..

[49] Reisin IL, Prat AG, Abraham EH, Amara JF, Gregory RJ, Ausiello DA, Cantiello HF (1994). The cystic fibrosis transmembrane conductance regulator is a dual ATP and chloride channel. J. Biol. Chem..

[50] Cantiello H (2001). Electrodiffusional ATP movement through CFTR and other ABC transporters. Pflügers Arch - Eur J Physiol.

[51] Hanrahan JW, Tabcharani JA (1990). Inhibition of an outwardly rectifying anion channel by HEPES and related buffers. J. Membr. Biol..

[52] Meineke B, Heimgärtner J, Lafranchi L, Elsässer SJ (2018). Methanomethylophilus alvus Mx1201 provides basis for mutual orthogonal Pyrrolysyl tRNA/Aminoacyl-tRNA synthetase Pairs in Mammalian Cells. ACS Chem. Biol..

[53] Cui Z, Mureev S, Polinkovsky ME, Tnimov Z, Guo Z, Durek T, Jones A, Alexandrov K (2017). Combining sense and nonsense codon reassignment for site-selective protein modification with unnatural amino acids. ACS Synth. Biol..

[54] Pott M, Schmidt MJ, Summerer D (2014). Evolved sequence contexts for highly efficient amber suppression with noncanonical amino acids. ACS Chem. Biol..

[55] Phillips-Jones MK, Watson FJ, Martin R (1993). The 3' codon context effect on UAG suppressor tRNA is different in Escherichia coli and human cells. J. Mol. Biol..

[56] Gerrits M, Budisa N, Merk H (2019). Site-specific chemoselective pyrrolysine analogues incorporation using the cell-free protein synthesis system. ACS Synth. Biol..

[57] Beránek V, Willis JCW, Chin JW (2019). An evolved Methanomethylophilus alvus Pyrrolysyl-tRNA Synthetase/tRNA pair is highly active and orthogonal in Mammalian Cells. Biochemistry.

[58] Schuler B, Eaton WA (2008). Protein folding studied by single-molecule FRET. Curr. Opin. Struct. Biol..

[59] Khushoo A, Yang Z, Johnson AE, Skach WR (2011). Ligand-driven vectorial folding of ribosome-bound human CFTR NBD1. Mol. Cell.

[60] Nishiguchi R, Tanaka T, Hayashida J, Nakagita T, Zhou W, Takeda H (2022). Evaluation of cell-free synthesized human channel proteins for in vitro channel research. Membranes.

