# Supplementary material for: Enhancing the performance of a mutant pyrrolysyl-tRNA synthetase to create a highly versatile eukaryotic cell-free protein synthesis tool
Source: Sci Rep. 2023 Sep 14;13:15236. doi: 10.1038/s41598-023-42198-8 (PMC10502014; doi:10.1038/s41598-023-42198-8)
Supplement: Supplementary file 1 — Supplementary Figures. [file 41598_2023_42198_MOESM1_ESM.pdf]

## Supplementary Information

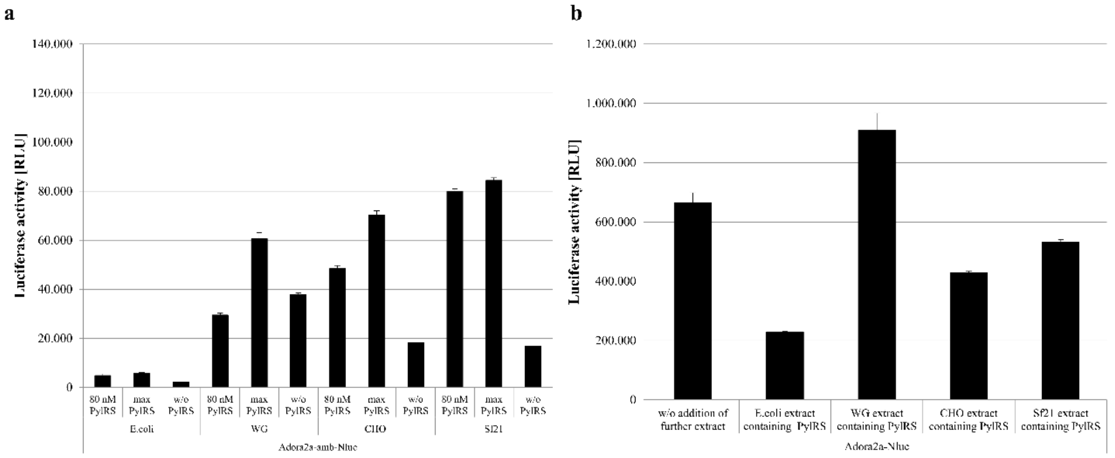


**Figure S1: Detection of amber-suppression efficiency in cell-free protein synthesis based on *Sf*21 lysate.** a) a) PylRS-AF was synthesized in cell-free reactions based on *E.coli*, wheat germ (WG), *Sf*21 and CHO in a CECF format. The supernatant of these reactions was supplemented to a *Sf*21 batch based cell-free reaction containing Adora2a-amb-Nluc as template. In the presence of active PylRS the full length protein with C-terminal luciferase was obtained. Nluc activity was measured after three hours synthesis reaction. The maximal possible PylRS-AF concentration and equal enzyme concentrations (80 nM) were added to the batch based reaction. b) The same supernatant described in a) was supplemented to a *Sf*21 based cell-free reaction containing Adora2a-Nluc to determine the effect of supernatants to the translation reaction. Nluc activity was measured after three hours synthesis reaction. Measurements were performed in duplicate. Data are shown as mean ± SD.

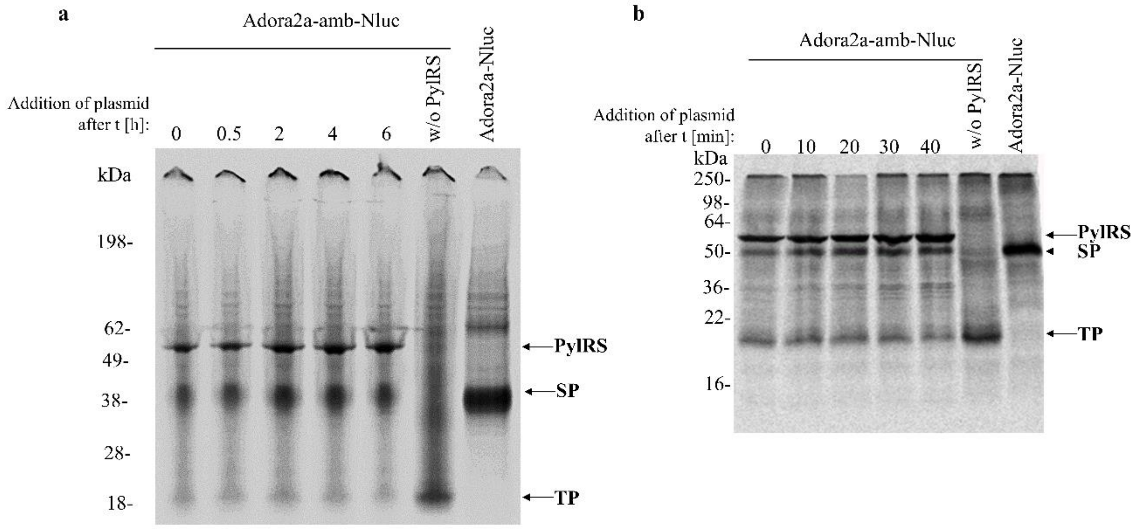


**Figure S2: Autoradiography of cell-free synthesized Adora2a variants corresponding to the Nluc assay shown in figure 4.** a) Autoradiography corresponding to the CECF reaction based on *Sf*21 cell lysate. b) Autoradiography corresponding to the batch reaction based on *Sf*21 cell lysate. SP: Suppression product. TP: Termination product.

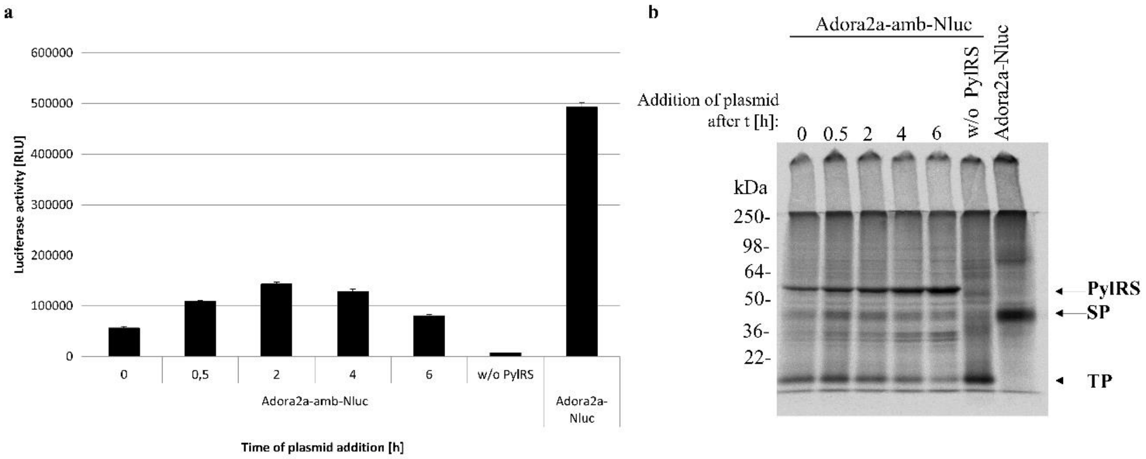


**Figure S3: Luciferase activity of co-expressed plasmids in CHO CECF cell-free systems.** PylRS-AF was co-expressed with Adora2a-amb-Nluc. The PylRS template was directly added at the beginning of the reaction. The Adora2a-amb-Nluc template was added after the indicated time. a) CECF-reaction reaction showing the successful synthesis of full length protein in the presence of PylRS. The addition of the Adora2a-amb-Nluc template between 2 and 4 hours resulted in the highest suppression efficiency. b) Autoradiography corresponding to the Nluc-assay showing similar results. Measurements were performed in duplicate. Data are shown as mean ± SD. SP: Suppression product. TP: Termination product.

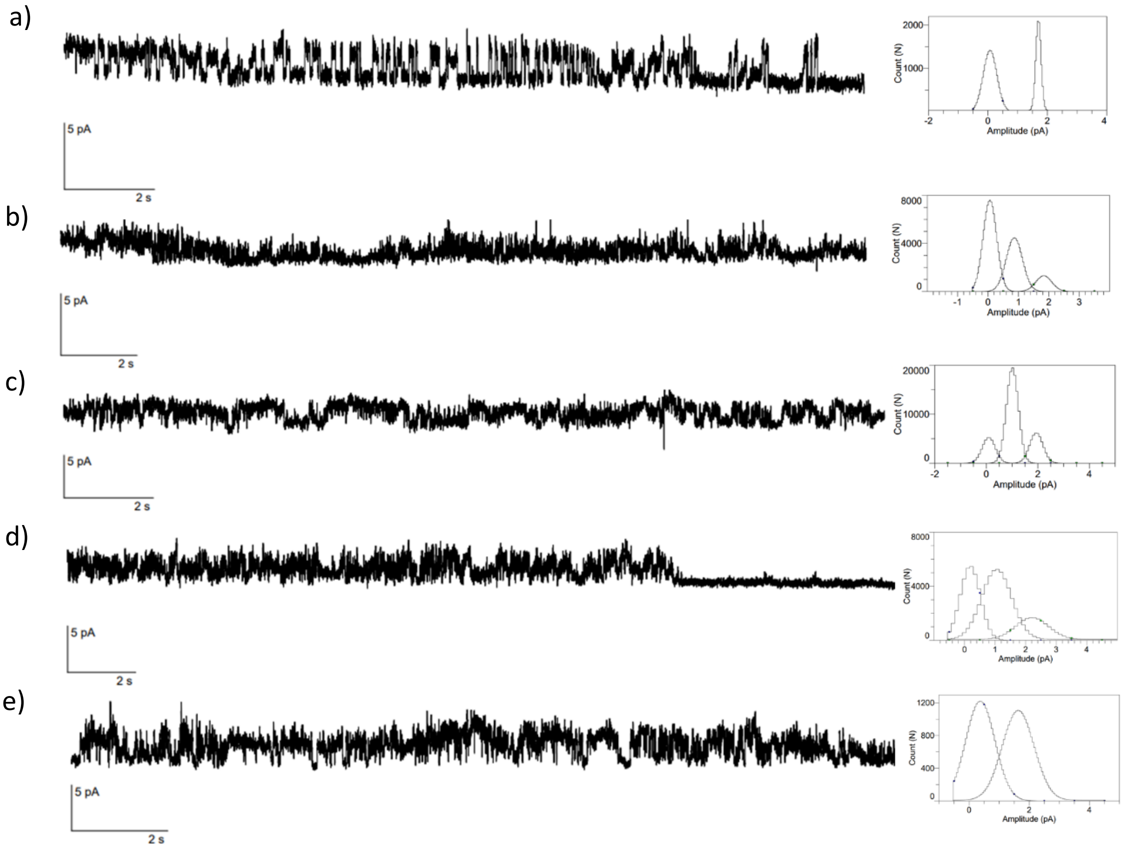

**Figure S4: Single-channel traces of CFTR with PylRS from different experiments recorded from the DPhPC planar lipid bilayers.** All experiments were performed in the presence of 10 mM HEPES, 150 mM NaCl, pH 7.0, in the presence of 8-12 mM ATP and 30-62.5 U /ml PKA.

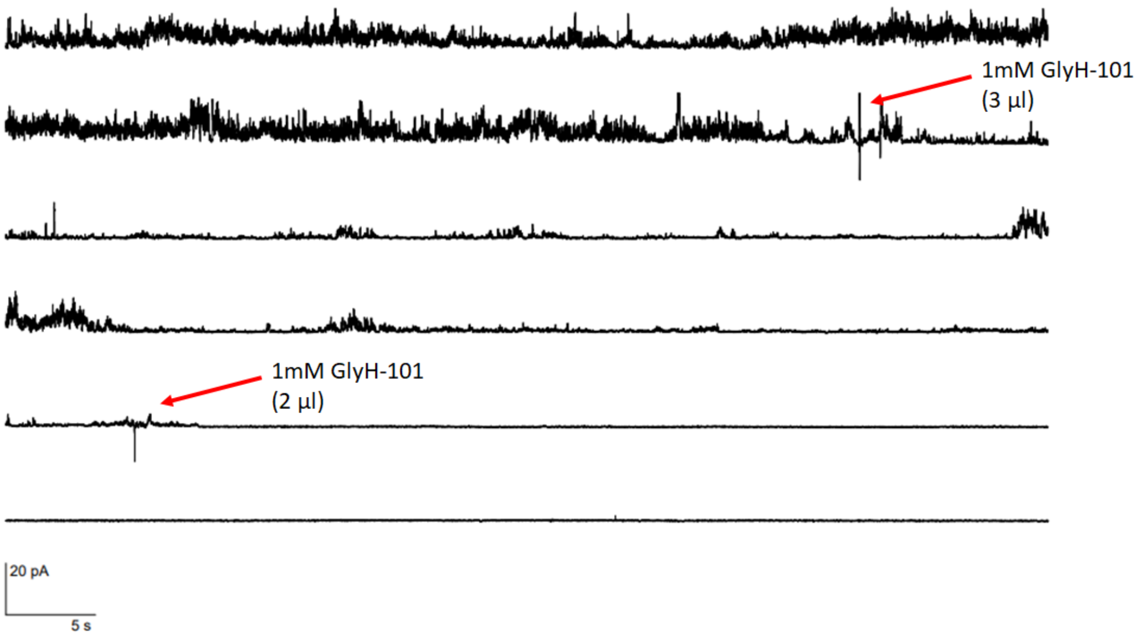


**Figure S5: Single-channel traces of CFTR-ambF157 with PylRS recorded from the DPhPC planar lipid bilayer.** Red arrows indicates the addition of 1 mM CFTR specific blocker GlyH-101. All experiments were performed in the presence of 10 mM HEPES, 150 mM NaCl, pH 7.0, in the presence of 8-12 mM ATP and 30-62.5 U/ml PKA.

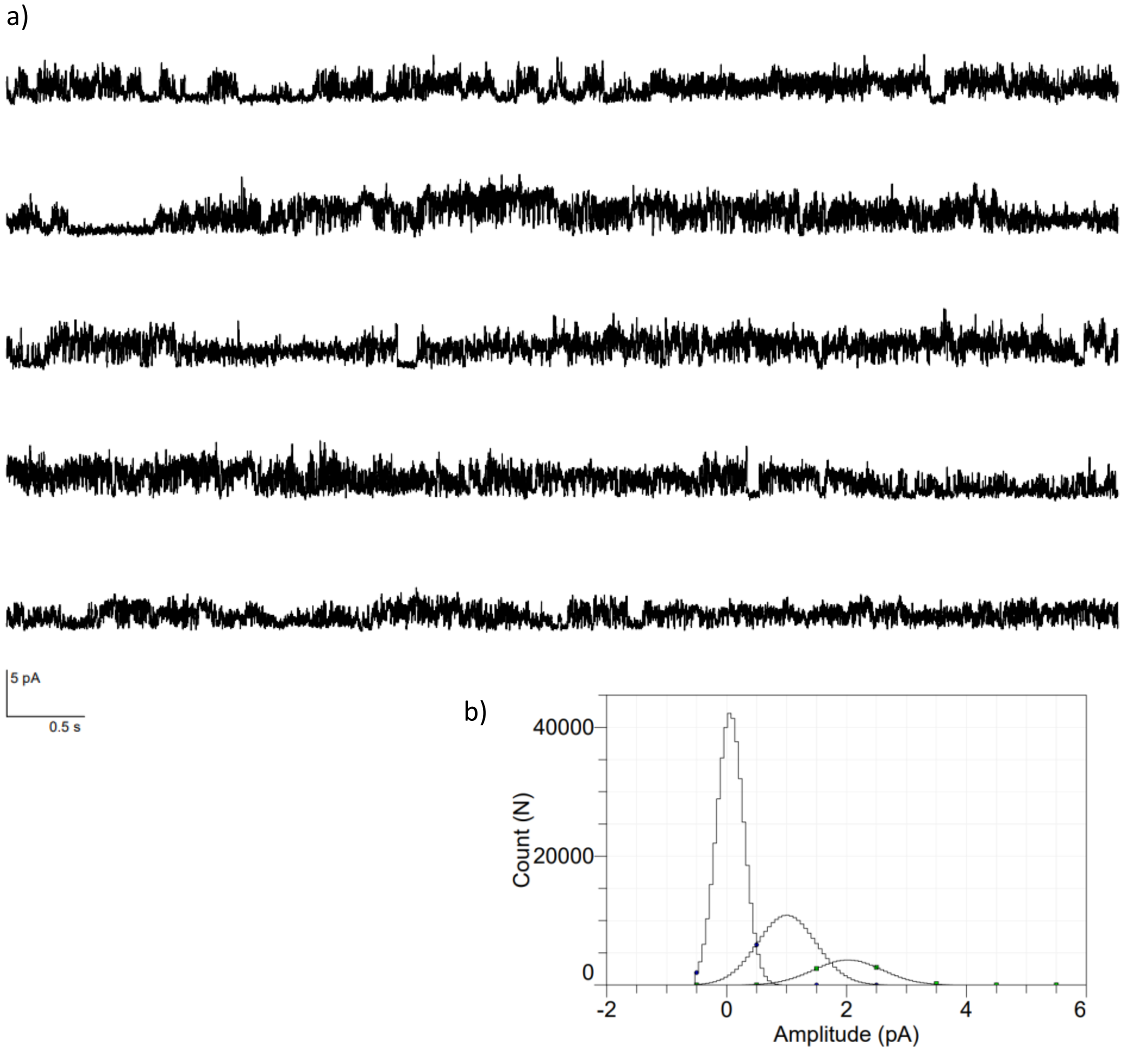


**Figure S6: Planar bilayer measurements of the CFTR-ambF157 with PylRS channel function.** a) Single-channel activity recordings from CFTR-ambF157 with PylRS incorporated microsomes reconstituted into planar DPhPC lipid bilayers at +100 mV. b) All point histogram of the corresponding trace showing three different levels corresponding to open (1 and 2 pA) and closed (0 pA) transient states of the ion channel. All recordings are made under 10 mM HEPES, 150 mM NaCl, pH 7.0, in the presence of 8-12 mM ATP and 30-62.5 U/ml PKA.

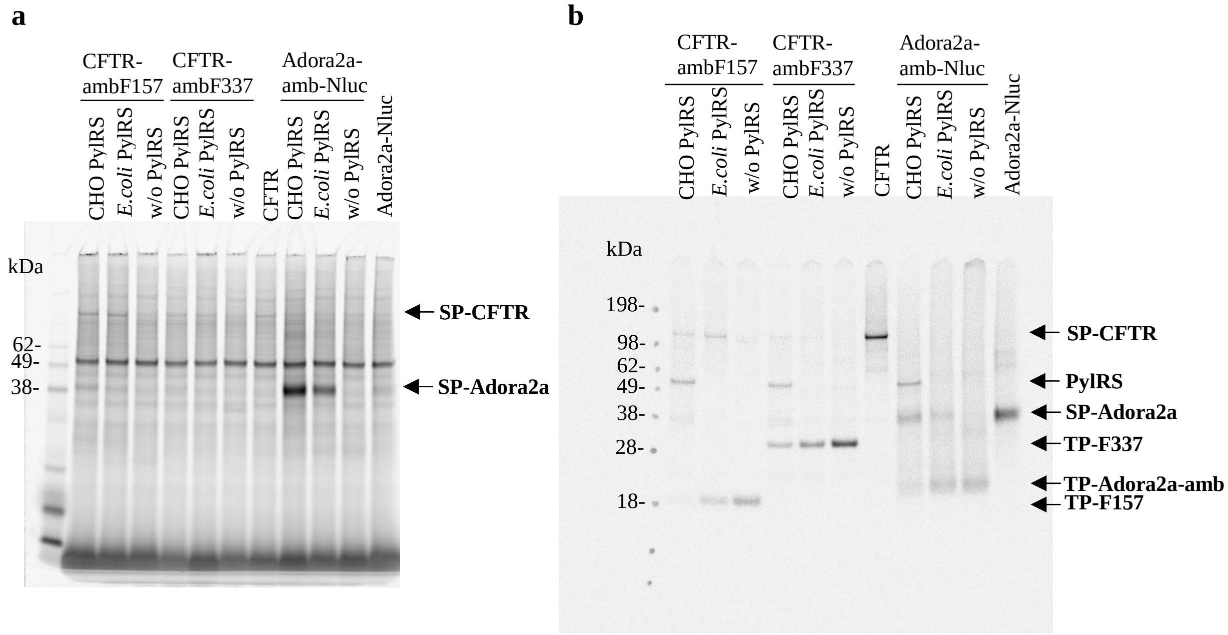


**Figure S7: Full and unedited images of Figure 3a, 3b, 5a and 5b. Site-specific fluorescent labeling of CFTR and Adora2a by PylRS-AF in *Sf*21 lysate.** Batch reactions in *Sf*21 lysate were carried out with CFTR-ambF157 and –F337 and Adora2a-amb-Nluc. Strained cyclooct-2-yn-1-methylcarbamate lysine (SCO) was added in combination with 2 µl CHO PylRS or 5 µM purified *E.coli* PylRS and tRNACUAPyl. After three hours the microsomal fraction was incubated with H-Tetrazine-Cy5. Afterwards the samples were precipitated by acetone and loaded on a NuPAGE™ 10% Bis-Tris protein gel. Fluorescence was detected by excition with a 633 nm laser. a) Fluorescent image; b) autoradiograph. Protein ladder: SeeBlue™ Plus2 Pre-stained Protein Standard. SP: Suppression product; TP: termination product

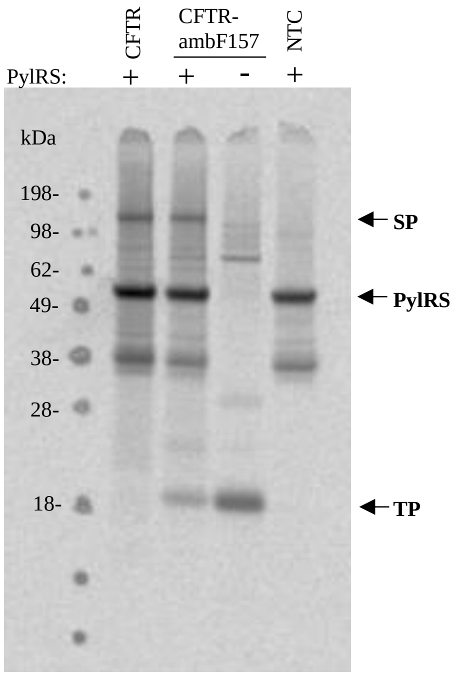

**Figure S8: Full and unedited image of Figure 5c. Site-specific incorporation of SCO into CFTR.** The non-canonical amino acid SCO was incorporated into CFTR-ambF157 during a *Sf*21 CECF based cell-free reaction in the presence or absence of PylRS-AF. The image shows the autoradiography. SP: Suppression product. TP: Termination product.

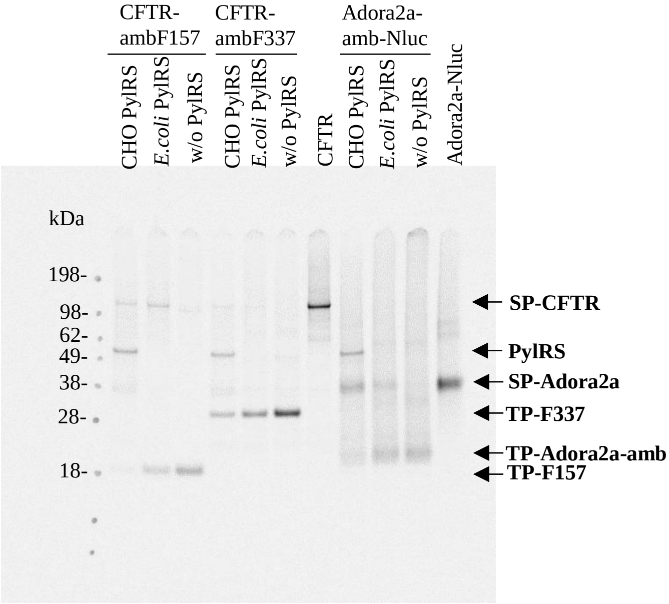


| | Band No | Band Volume | Protein | Suppression efficiency (%) |
|---|---|---|---|---|
| Lane 1 | 1 | 64927 | PylRS-AF | 23,25 |
| Lane 1 | 2 | 126173 | SP-Adora2a-amb-Nluc | |
| Lane 1 | 3 | 28320 | TP-Adora2a-amb-Nluc | |
| Lane 2 | | | | |
| Lane 3 | 1 | 142981 | TP-Adora2a-amb-Nluc | |
| Lane 4 | 1 | 542594 | Adora2a-amb-Nluc | |

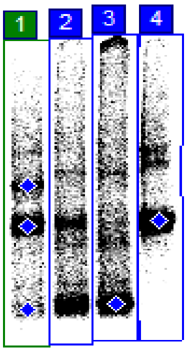


**Figure S9: Semi-quantitative analysis of Adora2a-amb-Nluc suppression efficiency by analysis of the autoradiograph.** Autoradiograph based on Supplementary figure S7b (unmodified version) was analyzed using ImageQuantTL software (GE Healthcare Life Sciences) utilizing the last 4 lanes to analyze the suppression efficiency of the Adora2a-amb-Nluc cell-free protein synthesis. Each lane shows the measured band volume for indicated protein bands (blue). Band numbering is from top to bottom. The suppression efficiency (%) is expressed as band volume of SP-Adora2a-amb-Nluc of Lane 1 band no 2 divided by band volume of Adora2a-amb-Nluc of lane 4 band no 1 and multiplied by 100.

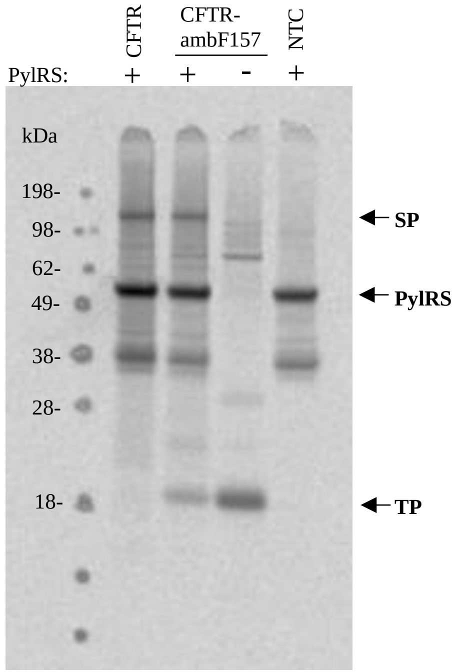


| | Band No | Band Volume | Protein | Suppression efficiency (%) |
|---|---|---|---|---|
| Lane 1 | 1 | 734596 | CFTR | 61,95 |
| Lane 1 | 2 | 10948309 | PylRS-AF | |
| Lane 1 | 3 | 1261617 | PylRS-AF degradation | |
| Lane 2 | 1 | 455110 | SP-CFTR-amb-F157 | |
| Lane 2 | 2 | 6449124 | PylRS-AF | |
| Lane 2 | 3 | 427677 | PylRS-AF degradation | |
| Lane 2 | 4 | 79646 | TP-CFTR-amb-F157 | |
| Lane 3 | 1 | 1220057 | TP-CFTR-amb-F157 | |
| Lane 4 | 1 | 3696535 | PylRS-AF | |
| Lane 4 | 2 | 463657 | PylRS-AF degradation | |

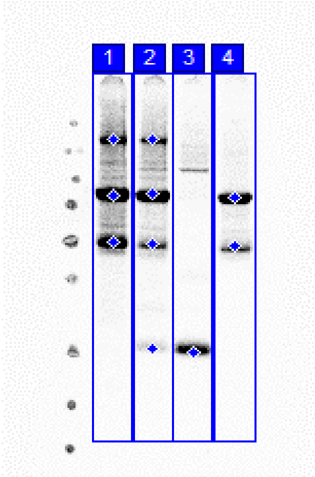


**Figure S10: : Semi-quantitative analysis of CFTR-amb-F157 suppression efficiency by analysis of the autoradiograph.** Autoradiograph based on Supplementary figure S8 (unmodified version) was analyzed using ImageQuantTL software (GE Healthcare Life Sciences) to analyze the suppression efficiency of CFTRa-mbF157 cell-free protein synthesis. Each lane shows the measured band volume for indicated protein bands (blue). Band numbering is from top to bottom. The suppression efficiency (%) is expressed as band volume of TP-CFTR-amb-F157 of Lane 2 band no 1 divided by band volume of CFTR of lane 1 band no 1 and multiplied by 100.

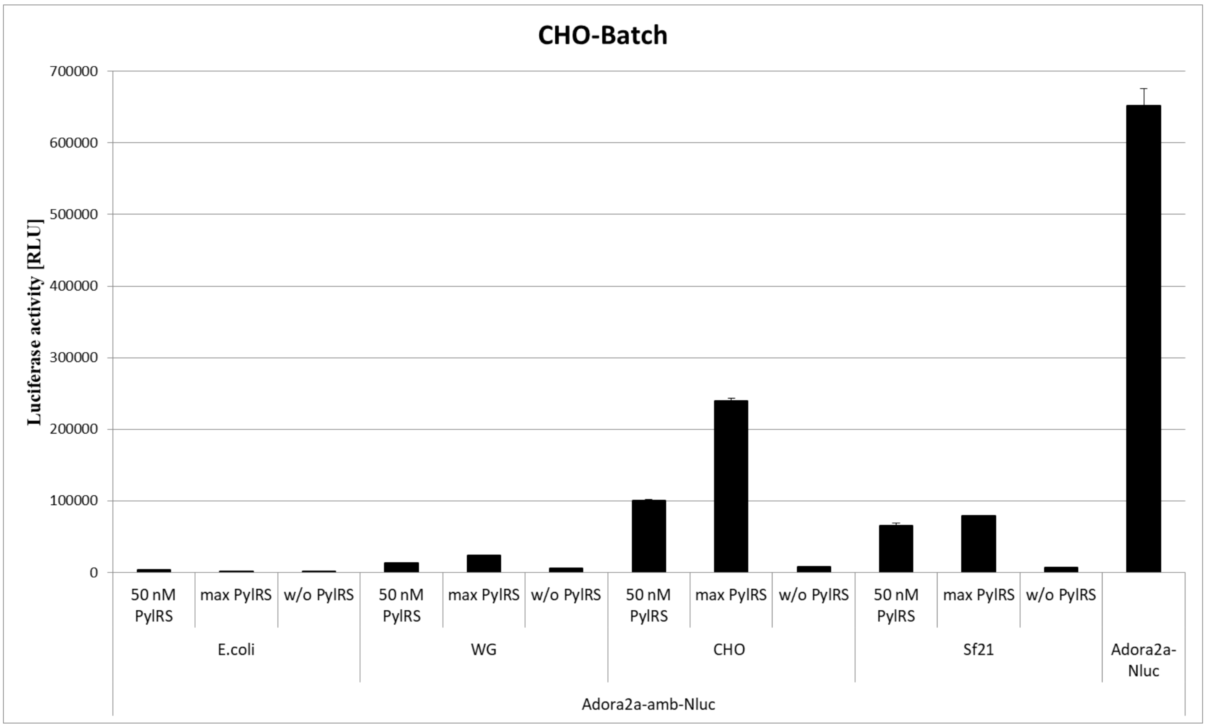


**Figure S11: Detection of amber-suppression efficiency in cell-free protein synthesis based on CHO lysate.** PylRS-AF was synthesized in cell-free reactions based on E.coli, wheat germ (WG), *Sf*21 and CHO in a CECF format. The translation mix of these reactions was supplemented to a CHO batch based cell-free reaction containing Adora2a-amb-Nluc as template. Nluc activity was measured after three hours of the CHO batch based cell-free reaction harboring different translation mixes containing PylRS-AF. The maximal possible PylRS-AF concentration in a batch based reaction and equal enzyme concentrations (50 nM) were added. Supplemented template: a) Adora2a-amb-Nluc. b) Adora2a-Nluc. Measurements were performed in technical replicates. Data are shown as mean ± SD.
